# Medical Institute of Louisville

**Published:** 1844-08

**Authors:** 


### MEDICAL INSTITUTE OF LOUISVILLE.

We are requested to call the attention of students of medicine to the fact, that but *one introductory* lecture is now delivered annually in the Medical Institute of Louisville, and that the regular didactic lectures are begun the following day. By this innovation nearly an entire week is gained. It is the determination of the Faculty to adhere to this arrangement, which was first introduced last season.

